# Air Pollution Exposure Impairs Airway Epithelium IFN-β Expression in Pre-School Children

**DOI:** 10.3389/fimmu.2021.731968

**Published:** 2021-10-18

**Authors:** Matteo Bonato, Elisa Gallo, Martina Turrin, Erica Bazzan, Federico Baraldi, Marina Saetta, Dario Gregori, Alberto Papi, Marco Contoli, Simonetta Baraldo

**Affiliations:** ^1^ Respiratory Diseases Clinic, Department of Cardiac, Thoracic, Vascular Sciences and Public Health, University of Padova, Padova, Italy; ^2^ Unit of Biostatistics, Epidemiology and Public Health, Department of Cardiac, Thoracic, Vascular Sciences and Public Health, University of Padova, Padova, Italy; ^3^ Respiratory Section, Department of Translational Medicine, University of Ferrara, Ferrara, Italy

**Keywords:** asthma, air pollution, interferon, PM10 (particulate matter <10 microns), NO_2_

## Abstract

**Introduction:**

Air pollution is a risk factor for respiratory infections and asthma exacerbations. We previously reported impaired Type-I and Type-III interferons (IFN-β/λ) from airway epithelial cells of preschool children with asthma and/or atopy. In this study we analyzed the association between rhinovirus-induced IFN-β/λ epithelial expression and acute exposure to the principal outdoor air pollutants in the same cohort.

**Methods:**

We studied 34 children (17asthmatics/17non-asthmatics) undergoing fiberoptic bronchoscopy for clinical indications. Bronchial epithelial cells were harvested by brushing, cultured and experimentally infected with Rhinovirus Type 16 (RV16). RV16-induced IFN-β and λ expression was measured by quantitative real time PCR, as was RV16vRNA. The association between IFNs and the mean exposure to PM10, SO2 and NO_2_ in the day preceding bronchoscopy was evaluated using a Generalized Linear Model (GLM) with Gamma distribution.

**Results:**

Acute exposure to PM10 and NO_2_ was negatively associated to RV16-induced IFNβ mRNA. For each increase of 1ug/m^3^ of NO_2_ we found a significative decrease of 2.3x10^3^ IFN-β mRNA copies and for each increase of 1ug/m^3^ of PM10 a significative decrease of 1x10^3^ IFN-β mRNA copies. No significant associations were detected between IFN-λ mRNA and NO_2_ nor PM10. Increasing levels of NO_2_ (but not PM10) were found to be associated to increased RV16 replication.

**Conclusions:**

Short-term exposure to high levels of NO_2_ and PM10 is associated to a reduced IFN-β expression by the airway epithelium, which may lead to increased viral replication. These findings suggest a potential mechanism underlying the link between air pollution, viral infections and asthma exacerbations.

## Introduction

Air pollution is increasingly recognized as an important cause of asthma exacerbations and, possibly, as a cofactor for asthma origins (1). Many studies have shown that the exposure to pollutants is associated to increased risk of respiratory viral infections, i.e. the most frequent triggers of asthma exacerbations, including rhinovirus, influenza and respiratory syncytial virus. In asthma patients there is evidence that the exposure to pollutants in the week preceding a respiratory viral infection increases the severity of the associated asthma exacerbations (2). The mechanisms by which air pollution may increase susceptibility to infections and favor viral-induced exacerbations in asthma patients are still largely unknown.

Previous studies- though not unanimously- have identified impaired antiviral immune response as a possible mechanism for increased susceptibility to infections in asthmatic patients. In particular, we previously reported, impaired type I (IFN-β) and type III interferon (IFN-λ) production following rhinovirus-16 (RV16) infection in bronchial epithelial cells of pre-school asthmatic and/or atopic children compared to non-asthmatic non-atopic children (3). Furthermore, we reported that such impaired IFN response was associated to persistence of asthma symptoms up to school-age (4) in line with the epidemiological data suggesting that respiratory tract infections in childhood increase the risk of asthma.

To the best of our knowledge, no study has ever investigated the association between viral-induced IFN responses in bronchial epithelial cells and exposure to air pollution in children at risk for asthma.

In this study we sought to investigate the relationship between exposure to air pollutants (nitrogen dioxide-NO_2_, particulate matter less than 10 µm in aerodynamic diameter-PM10, sulfur dioxide-SO_2_) and RV16-induced IFN-β and λ expression in primary bronchial epithelial cells obtained from a cohort of pre-school children (3). Aim of this study is to evaluate whether RV16 induced IFN-β and λ mRNA levels were affected by the ambient air concentration of: i) nitrogen dioxide-(NO_2_), ii) particulate matter less than 10 µm in aerodynamic diameter (PM10) and iii) sulfur dioxide (SO_2_).

## Methods

### Study Population

The study design and the population of the children cohort considered in this study has been previously described in details (3). The study was performed according to the Declaration of Helsinki and was approved by the Ethics Committee of the Padova City Hospital. Children underwent bronchoscopy, with bronchoalveolar lavage, for appropriate clinical indications according to current guidelines (5). Bronchial biopsy and brushing were harvested for research purposes during bronchoscopy upon approval from the children’s parents. A detailed medical history was collected by a respiratory pediatrician before bronchoscopy. The physician administered parental interviews investigating the pattern of respiratory symptoms, the frequency of respiratory tract infections (RTI) in the previous year and on-course treatment. Presence of wheezing with a pattern suggestive of asthma was based on the report of repeated episodes of wheezing in the previous year, often associated to cough and dyspnea, particularly at night or in the early morning, that was responsive to bronchodilators. None of the children in the non-asthmatic control group complained of episodes of wheezing, breathlessness or cough that were responsive to bronchodilators. All children underwent routine blood tests, including complete white blood cell count (total leukocytes, neutrophils, lymphocytes, monocytes, eosinophils and basophils) and total/specific IgE. The presence of atopy was defined by an increase of total (higher than the age-related normal levels) and specific IgE (>0.35KU/L; IMMunoCAp, Phadia, Sweden). In particular, specific IgE for the following aeroallergens were investigated in all children: house dust mite (*Dermatophagoides pteronyssinus and Dermatophagoides farinae*), moulds (*Alternaria alternate*), cat dander and grass pollens (*Lolium perenne, Poa pratensis, Phleum pratense, Dactylis glomerata and Cynodon dactylon*).

### Air Pollution Exposure Evaluation

Daily levels of PM10, NO_2_ and SO_2_ were retrieved from the monitoring stations of the Environmental Protection and Prevention Agency of Veneto Region (ARPAV) (6, 7). The concentrations of the pollutant the day of the bronchoscopy and the day before were collected. Each child was linked to the data of the monitoring station nearest to his/her residence. Schools were in proximity of the residential address for all children. Air pollution concentrations were compared with the 2005 WHO air quality guidelines, which set cut-offs not to be exceeded at 50 μg/m^3^ for PM10 and 40 μg/m^3^ for NO_2_ (8).

### Primary Bronchial Epithelial Cell Cultures for Detection of IFN-β, IFN-λ, and Viral RNA

Primary bronchial epithelial cells (HBEC) were harvested by bronchial brushing, handled and cultured as previously detailed (3). Briefly, HBEC cultures were set up into hormonally supplemented bronchial epithelial growth medium (BEGM, Clonetics, San Diego, USA) containing 50U/ml penicillin and 50 mg/ml streptomycin. At 80% confluency, cells were resuspended, counted (1.8 x10^5^ cells/ml) and immediately seeded in 12-well plates (2 ml in each well) for the infection. Cells were starved in bronchial epithelial basal medium (BEBM with no supplements) overnight before exposure to RV16. Rhinovirus type 16 (RV16; a major group rhinovirus) was obtained from the Health Protection Agency Culture Collections, Health Protection Agency Microbiology Services, Salisbury, United Kingdom. The virus was used at a multiplicity of infection of 5 for all experiments. Cells were exposed to rhinovirus at room temperature for 1 hour, washed with phosphate buffered saline (PBS) to eliminate unbound virus and grown in BEGM (for 8hrs or 48hrs). Samples from each donor were performed in duplicate. As internal control of rhinovirus infection, we exposed the cell cultures to inocula from which virus particles were removed by ultrafiltration (Amikon, London, UK) or inactivated by UV-irradiation.

Quantitative real-time PCR was carried out for rhinovirus, IFN-β, IFN-λ, viral RNA (vRNA) and18S rRNA (a housekeeping gene) at 8 hrs post infection, as previously described (3). Interferons and viral RNA (vRNA) expressions were normalized to 18S rRNA levels, compared with standard curves, and expressed as copy numbers per microgram of RNA. Furthermore, detection of IFN-β and IFN-λ protein (and IL-8 as internal control) was performed at 48hrs after infection as previously described (3). Since 8 hrs cultures for mRNA detection were prioritized, and only when enough cells were available plates for the 48 hrs time point were set-up, IFN-β and IFN-λ protein levels were available in a subset of children in our cohort (17 subjects for IFN-β, 9 subjects for IFN-λ). Due to the smallness of the sample we failed to obtain a reliable association model between pollution exposure and protein production, so we did not consider it for further analyses.

### Statistical Analysis

Children’s characteristics were expressed as median and IQR for continuous variables, and counts and percentages for categorical variables. The difference in explanatory variables was assessed using a Chi-squared test or Fisher test for dichotomous and categorical variables, or Mann–Whitney U test for continuous variables.

The acute effect of air pollution on RV16 vRNA copies, IFN-β and IFN-λ mRNA copies was modeled using a Generalized Linear Model (GLM) assuming a Gamma distribution with an identity link function to account for the skewness of the outcomes (9). Single pollutant models were performed including the acute exposure to the pollutant and the presence of asthma as covariates in the linear predictor. For what concerns acute exposure, the average concentration of the pollutant between the day of the bronchoscopy and the day before (lag 01) was used.

In view of the limited sample size and the high degree of skewness in the covariates, to evaluate stability of the results, a sensitivity analysis was conducted by replacing the variable indicating presence of asthma with the one representing the presence of atopy (10, 11). All analyses were performed using R statistical software (12, 13).

## Results

Patients’ characteristics were previously detailed (3). Briefly, the cohort included 17 asthmatics (either atopic: n=8 or non-atopic: n=9) and 17 controls (8 atopic; 9 non-atopic). The bronchoscopic procedure was well tolerated by all children, and no complications were encountered. Clinical indications for bronchoscopy did not differ significantly between asthmatic and non-asthmatic children. Asthmatic and non-asthmatic children had a similar age and gender distribution (**Table 1**). No children was chronically treated with oral or high-dose inhaled corticosteroids. Asthmatic children showed higher levels of peripheral blood eosinophils (p=0.02), BAL eosinophils were not different in the two groups. The two groups of children had a comparable history of respiratory tract infections (RTI). Though asthmatic and non-asthmatic children were matched for atopic status (as per design of the original study), a non-significant trend for increased levels of total serum IgE was observed in asthmatic children compared to non-asthmatics (p=0.074).

**Table 1:** Description of the whole cohort and comparison between asthmatic and non-asthmatic children.

	WHOLE CHORT	ASTHMATICS	NON-ASTHMATICS	p
Subjects	34 (100%)	17 (50%)	17 (50%)	–
Male (n; %)	18 (52.9%)	11 (64.7%)	7 (41.2%)	0.169
Age (y)	5 [4-6]	5 [3.5-6.5]	5 [4-5.5]	0.973
Symptoms onset (y)	1 [0.5-3.5]	1 [0.5-3.5]	n.a.	–
RTI (ep/month)	1 [0-2]	2 [0.5-2.5]	2 [1-2]	0.786
ICS (n; %)	11 (32%)	10 (58.8%)	1 (5.9%)	0.001
Air pollution exposure PM10 (μg/m^3^) NO_2_ (μg/m^3^)	N=30 40.5 [23.0-53.0] 39.8 [28.9-47.5]	N=14 40.5 [27.5-59.1] 40.7 [32.2-50.4]	N=16 34.5 [22.6-46.2] 36.0 [24.0-46.9]	0.381 0.270
Serum IgE (kU/L)	47.5 [13.9-221.5]	89 [29-504]	32 [12.2-136]	0.079
Blood Eosinophils (cell/µL)	269 [142-539]	443 [220-710]	260 [74.5-361]	0.024
BAL Eosinophils (%)	0 [0-2.25]	0 [0-1.5]	0 [0-3.5]	0.483
Biopsy Eosinophils (cell/mm^2^)	14 [6.7-126.5]	63 [13-161]	12 [0-81]	0.217
Basement membrane thickness (µm)	4.66 [3.83-5.19]	5.19 [4.36-5.96]	4.05 [3.49-4.79]	0.032
IFN-β mRNA (x10^3^ copies/μg)	3.7 [1.0-77.6]	2.1 [0.8-16.8]	32.2 [2.9-213.3]	0.049
IFN-λ mRNA (x10^3^ copies/μg)	3.8 [0.8-21.3]	1.2 [0.4-6.5]	14.2 [1.4-142.5]	0.011
RV16 vRNA (x10^3^ copies/μg)	44.4 [12.3-542.1]	224.3 [35.5-772.6]	25.5 [9.8-47.5]	0.009

The p-value is referred to the Mann-Whitney-U test or Chi-square/Fisher’s exact comparison between asthmatic and non-asthmatic children. Data are expressed as median [interquartile range]. RTI, respiratory tract infections during previous year; ICS, inhaled corticosteroid therapy at bronchoscopy; n.a., not applicable.

**Figure 1:**
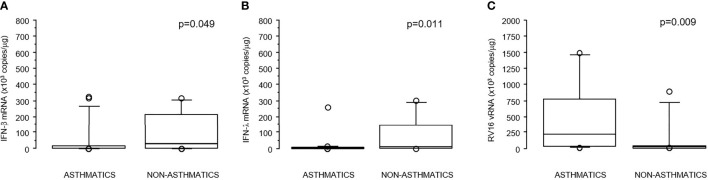
**(A–C)** Boxplots reporting values of IFN-λ mRNA **(A)**, IFN-β mRNA **(B)**, and RV16 vRNA **(C)** in asthmatics and non-asthmatics children. Bottom and top of the box-plot: 25^th^ and 75^th^ percentiles; solid line: median; brackets: 10° and 90° percentiles.

RV16-induced IFN-β induction in asthmatic children was significantly reduced compared to non-asthmatic children (p=0.049); a similar reduction was also observed for IFN-λ (p=0.011). The reduction in IFNs levels was mirrored by a significant increase in RV16 vRNA levels in asthmatic children compared to non-asthmatics (p=0.009) as previously described (**Table 1** and **Figures 1A**–**C**).

Data on air pollution were available for 30 out of 34 children. Data on median levels of outdoor air pollution on lag01 are summarized in **Table 1**. SO_2_ levels were virtually undetectable across the whole region so we did not consider this pollutant for further analyses. As shown in the table children in our cohort were exposed to high levels of air pollutants. The median exposure values for NO_2_ and PM10 were very close to the corresponding WHO recommended highest limit. Of note, we did not observe significant different levels of exposure to PM10 and NO_2_ among districts, nor between asthmatic and non-asthmatic children.

**Figure 2:**
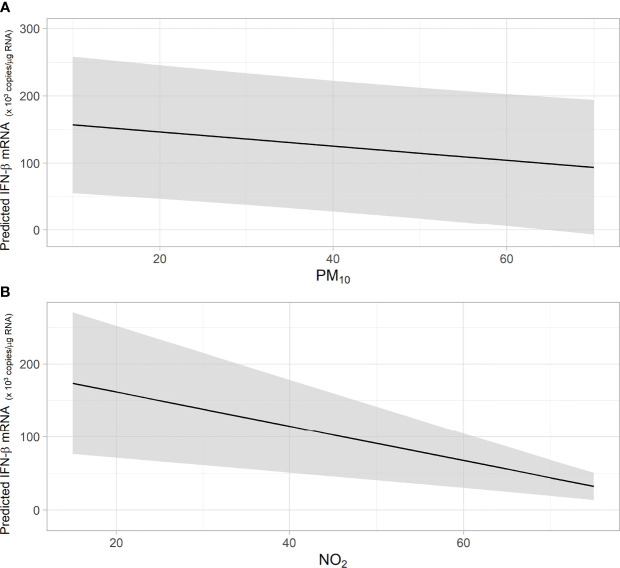
**(A, B)** The figures show the predicted values of IFN- β mRNA in association with short-term exposure to PM10 **(A)** and NO2 **(B)**. Shaded area indicates the confidence interval (CI). Predicted values of IFN- β mRNA were obtained from Generalized Linear Model (GLM).

When considering the whole cohort of preschool children, a negative association between acute (lag01) exposure to PM10 or NO_2_ and the levels of RV16-induced IFNβ mRNA in HBEC was observed (**Figure 2** and **Table 2**). For each increase of 1 ug/m^3^ of NO_2_ we found a significative decrease of 2.3 x10^3^ in RV16-induced IFN-β mRNA copy number. For each increase of 1ug/m^3^ of PM10 a significative decrease of 1 x10^3^ in RV16-induced IFN-β mRNA copies was observed (**Figures 2A, B**). No significant associations were detected between RV16-induced IFN-λ mRNA and NO_2_ nor PM10. Exposure to increasing levels of NO_2_ (but not PM10) was positively associated to increased RV16 replication in HBEC (**Table 2**).

**Table 2:** β coefficients and relative 95% Confidence Interval (CI) of the multivariable models.

Outcome	Pollutant	β	95% CI
IFN- β mRNA (x10^3^ copies/μg)	PM10	-1.05	-1.97; -0.14
	NO_2_	-2.36	-3.70; -1.01
IFN-λ mRNA (x10^3^ copies/μg)	PM10	1.04	-0.71; 2.79
	NO_2_	0.80	-0.99; 2.58
RV16 vRNA (x10^3^ copies/μg)	PM10	8.06	-13.10; 29.21
	NO_2_	31.73	6.59; 56.88

Since we have previously shown that both asthma and atopy were associated to reduced IFN expression by epithelial cells (3), we performed a sensitivity analysis in which the presence of asthma was replaced with the presence of atopy. Consistently, the analyses confirmed the significant decrease of IFN-β mRNA copies in association with both PM10 and NO_2_. However, the significance of the association between RV16 vRNA copies and NO_2_ was lost in this sensitivity analysis. We also confirmed null associations between RV16 replication and PM10, or between IFN-λ mRNA copies and PM10/NO_2_ (data not shown).

## Discussion

In this study we explored the relationship between exposure to air pollutants and innate immune response to viral infection in primary epithelial cells in a cohort of preschool asthmatic children and controls. We found that acute exposure to either NO_2_ and PM10 was associated to impaired antiviral immune response, in terms of RV16-induced IFN-β expression. Furthermore, exposure to NO_2_, but not PM10, was associated to increased levels of viral replication.

In our previous study performed in this cohort, we have demonstrated that the deficient expression of interferons by epithelial cells in response to rhinovirus, which was previously documented only in adults with atopic asthma, was present even in preschool children and that both asthma and atopy were independent factors associated to this reduced IFN induction. In this study we have considered the effect of air pollution on the innate immune response to viral infection.

An epidemiological link between air pollution and lower tract respiratory infections has been well described in multiple cohorts, either in developed and in emerging countries (14, 15). Nevertheless, the association between antiviral immune response and air pollutants has been investigated mainly systemically, correlating the concentration of air pollutants to serum levels of IFN-γ (15, 16). So far, the evidence relating air pollution to impairment of innate immune responses directly on the lung has been scarce. Our results indicate that exposure to increasing levels of air pollutants, was related to an impaired IFN-β expression from the airway epithelium, which resulted in increased viral replication. No such effect of PM10 and NO_2_ was observed on IFN-λ in our study, which points out the different regulation of type I and type III IFNs (17). To our knowledge, this is the first study that investigates *ex-vivo* the influence of air pollution on interferon responses from the airway epithelium in children.

Our findings would point to the mechanisms through which air pollution can negatively affect the biological integrity of airway epithelial barrier predisposing to respiratory infections. It is widely recognised that air pollutants may influence the expression of multiple genes through epigenetic modifications, such as gene methylation, particularly during intrauterine life and early infancy (18). Earlier studies have focussed on the regulation of IFNγ production by PBMC (19), while few data are available on the effect of pollution on IFN expression in the lung. In line with our results, Tao and coworkers reported *in vitro* that exposure to particulate matter (PM2.5) deteriorated influenza virus infection by reducing the production of IFN-β by alveolar macrophages (20). Mechanistically, PM 2.5 exposure did suppress the NLRP3 inflammasome and IL-1β through the AHR-TIPARP signaling pathway. The Aryl hydrocarbon receptor (AHR) is a transcription factor that mediates the toxic activity of many environmental xenobiotics and it has been shown that AHR, acting on the TCDD-inducible poly-ADP-ribose polymerase (TIPARP), is a negative regulator of type I interferon expression. Activation of AHR is able to upregulate expression of the ADP-ribosylase TIPARP, which in turn causes down-regulation of type I interferon responses (21).

The mechanisms regulating the interaction between the airway epithelium and air pollutants is a current gap in asthma research (22). Indeed, despite a recent statement from the American Thoracic Society concluded that there is enough epidemiologic evidence indicating a causal link between exposure to outdoor air pollution and asthma, the underlying pathophysiology has not been thoroughly studied (23). The appraisal of the role of air pollution should be addressed in the short term, as a cause of asthma exacerbations, but also in the long term as a possible cofactor for asthma persistence. Indeed, in a longitudinal follow-up of our cohort we were able to demonstrate that the abnormal IFN response in early childhood (mean age 5yrs) correlated with the persistence of asthma symptoms throughout adolescence (4). It is thus conceivable that air pollution, by downregulating innate immune responses in early life, may track its effect throughout adulthood.

Of interest, we recently observed a dual effect of long-term pollutants exposure on cellular inflammation in airway tissue: while in asthmatic children air pollution enhances eosinophilic inflammation in bronchial mucosa, in non-asthmatic ones it associates with reduced numbers of both eosinophils and neutrophils (24). Altogether the results of the two studies suggest that air pollution can have a pro-inflammatory effect in susceptible subjects (asthmatics) while, at the same time, impairing innate immune responses. We can envisage the existence of one or multiple immunological checkpoints (e.g. AHR) able to heighten or desensitize innate responses by integrating several inputs, either endogenous or exogenous (e.g. air pollution or the Type-2 cytokine environment) (25).

A major strength of our study is the well characterized cohort of children undergoing bronchoscopy with brushing collection. The study, however, has limitations: first, analyses are based on regionally available pollutants measurements, not on personal exposure, and are limited to RV16 stimulation. Second, the limited sample size of our cohort and the lack of extended temporal data on pollutants exposure, hampered the possibility to perform more complete analyses which could account for the high degree of heterogeneity and manage potential confounding covariates. Furthermore, since protein measurements were available only in a subset of subjects, we could not investigate the association between air pollution and IFNs proteins. Finally, we are conscious that our patient sample is not representative of the general population due to the clinical conditions for which bronchoscopy was indicated either in asthmatic and non-asthmatic children. However, lower airway sampling during bronchoscopy in a pediatric population only for research purposes would be ethically unacceptable in our Hospital setting. More readily available biological samples such as nasal epithelial cells could be explored in future studies as a possible alternative to airway biopsy specimens since they may reflect, at least in part, innate immune responses from lower airways (26).

In conclusion, we demonstrated that acute exposure to high levels of NO2 and PM10 is associated to a reduced expression of IFN-β by airway epithelium and, consequently, to increased viral replication. Our results suggest that such impaired IFN-β response could be a pivotal element of the mechanism underlying the epidemiological link between air pollution, respiratory infections susceptibility, and asthma development that deserves further investigation.

## Data Availability Statement

The raw data supporting the conclusions of this article will be made available by the authors, without undue reservation.

## Ethics Statement

The studies involving human participants were reviewed and approved by Ethics Committee of the Padova City Hospital. Written informed consent to participate in this study was provided by the participants’ legal guardian/next of kin.

## Author Contributions

MB, EG, AP, MC, MS, SB: conception of the study design, drafting and revision of the manuscript. MB, EG, MT, EB, FB, DG, SB: acquisition and analysis of data (bronchoscopy, clinical examination, experimental analyses on bronchial biopsies; statistical analysis).

## Funding

University of Padova (BARA_BIRD2020_01).

## Conflict of Interest

MS reports grants from Chiesi Farmaceutici, grants from Laboratori Guidotti, grants from Bottega Veneta, grants from University of Padova- Italy, outside the submitted work. AP reports grants, personal fees, non-financial support and other from GlaxoSmithKline, grants, personal fees and non-financial support from AstraZeneca, grants, personal fees, non-financial support and other from Boehringer Ingelheim, grants, personal fees, non-financial support and other from Chiesi Farmaceutici, grants, personal fees, non-financial support and other from TEVA, personal fees, non-financial support and other from Mundipharma, personal fees, non-financial support and other from Zambon, personal fees, non-financial support and other from Novartis, grants, personal fees and non-financial support from Menarini, personal fees, non-financial support and other from Sanofi/Regeneron, personal fees from Roche, grants from Fondazione Maugeri, grants from Fondazione Chiesi, personal fees from Edmondpharma, outside the submitted work. MC reports grants, personal fees and non-financial support from Chiesi, personal fees and non-financial support from AstraZeneca, personal fees and non-financial support from Boehringer Ingelheim, personal fees and non-financial support from Alk-Abello, grants, personal fees and non-financial support from GlaxoSmithKline, personal fees and non-financial support from Novartis, personal fees and non-financial support from Zambon, grants from University of Ferrara - Italy, outside the submitted work.

The remaining authors declare that the research was conducted in the absence of any commercial or financial relationships that could be construed as a potential conflict of interest.

## Publisher’s Note

All claims expressed in this article are solely those of the authors and do not necessarily represent those of their affiliated organizations, or those of the publisher, the editors and the reviewers. Any product that may be evaluated in this article, or claim that may be made by its manufacturer, is not guaranteed or endorsed by the publisher.

## Abbreviations

AHR, Aryl hydrocarbon receptor; ARPAV, Environmental Protection and Prevention Agency of Veneto Region; BAL, Bronchoalveolar lavage; BEBM, bronchial epithelial basal medium; BEGM, bronchial epithelial growth medium; HBEC, primary bronchial epithelial cells; ICS, inhaled corticosteroids; IgE, immunoglobulin E; IFN, interferon; NO_2_, nitrogen dioxide; mRNA, messenger RNA; PCR, polymerase chain reaction; PBS, phosphate buffered saline; PM10, particulate matter less than 10 µm in aerodynamic diameter; RNA, ribonucleic acid; RSV, respiratory syncytial virus; RTI, respiratory tract infections; RV16, rhinovirus type 16; SO_2_, sulfur dioxide; TIPARP, TCDD-inducible poly-ADP-ribose polymerase; vRNA, viral RNA; WHO, world health organization.

## References

[1] Guarnieri M Balmes JR. Outdoor Air Pollution and Asthma. Lancet (2014) 383:158192. doi: 10.1016/S0140-6736(14)60617-6 PMC4465283 24792855

[2] Chauhan AJ Inskip HM Linaker CH Smith S Schreiber J Johnston SL. Personal Exposure to Nitrogen Dioxide (NO2) and the Severity of Virus-Induced Asthma in Children. Lancet (2003) 361:1939–44. doi: 10.1016/S0140-6736(03)13582-9 PMC7112409 12801737

[3] Baraldo S Contoli M Bazzan E Turato G Padovani A Marku B. Deficient Antiviral Immune Responses in Childhood: Distinct Roles of Atopy and Asthma. J Allergy Clin Immunol (2012) 130:1307–14. doi: 10.1016/j.jaci.2012.08.005 22981791

[4] Baraldo S Contoli M Bonato M Snijders D Biondini D Bazzan E. Deficient Immune Response to Viral Infections in Children Predicts Later Asthma Persistence. Am J Respir Crit Care Med (2018) 197:673–5. doi: 10.1164/rccm.201706-1249LE 28862881

[5] Faro A Wood RE Schechter MS Leong B Wittkuge lE Abode K. Official American Thoracic Society Technical Standards: Flexible Airway Endoscopy in Children. Am J Respir Crit Care Med (2015) 191:1066–80. doi: 10.1164/rccm.201503-0474ST 25932763

[6] European Parliament and the Council of the European Union. Directive 2008/50/EC of the European Parliament and of the Council of 21 May 2008 on ambient air quality and cleaner air for Europe. Off J Eur Union (2008) 152:1–44.

[7] Gallo E Folino F Buja G Zanotto G Bottigliengo D Comoretto R. Iliceto S Daily Exposure to Air Pollution Particulate Matter Is Associated With Atrial Fibrillation in High-Risk Patients. Int J Environ Res Public Health (2020) 17:6017. doi: 10.3390/ijerph17176017 PMC7504134 32824908

[8] Air Quality Guidelines. Global Update 2005. Particulate Matter, Ozone, Nitrogen Dioxide and Sulfur Dioxide . Available at: https://www.euro.who.int/en/health-topics/environment-and-health/Housing-and-health/publications/pre-2009/air-quality-guidelines.-global-update-2005.-particulate-matter,-ozone,-nitrogen-dioxide-and-sulfur-dioxide (Accessed Aug 5, 2020).

[9] Hastie TJ Tibshirani RJ. Generalized Additive Models, 1 Edition. Boca Raton, Fla: Chapman and Hall/CRC (1990).

[10] Burnham KP Anderson DR. Model Selection and Multimodel Inference: A Practical Information-Theoretic Approach. 2nd Edn. New York: Springer-Verlag (2002).

[11] Zhang Z. Multiple Imputation for Time Series Data With Amelia Package. Ann Transl Med (2016) 4:56. doi: 10.3978/j.issn.2305-5839.2015.12.60 26904578 PMC4740012

[12] Honaker J King G Blackwell M. Amelia: A Program for Missing Data (2019). Available at: https://CRAN.R-project.org/package=Amelia (Accessed July 21, 2020).

[13] Wood S. Mgcv: Mixed GAM Computation Vehicle With Automatic Smoothness Estimation (2019). Available at: https://CRAN.R-project.org/package=mgcv (Accessed July 21, 2020).

[14] HEI Collaborative Working Group on Air Pollution, Poverty, and Health in Ho Chi Minh City Le TG Ngo L Mehta S Do VD Thach TQ. Effects of Short-Term Exposure to Air Pollution on Hospital Admissions of Young Children for Acute Lower Respiratory Infections in Ho Chi Minh City, Vietnam. Res Rep Health Eff Inst (2012) 169:5–72. 22849236

[15] Ciencewicki J Jaspers I. Air Pollution and Respiratory Viral Infection. Inhal Toxicol (2007) 19:1135–46. doi: 10.1080/08958370701665434 17987465

[16] Klümper C Krämer U Lehmann I von Berg A Berdel D Herberth G. Air Pollution and Cytokine Responsiveness in Asthmatic and non-Asthmatic Children. Environ Res (2015) 138:381–90. doi: 10.1016/j.envres.2015.02.034 25769127

[17] Lazear HM Schoggins JW Diamond MS. Shared and Distinct Functions of Type I and Type III Interferons. Immunity (2019) 50:907–23. doi: 10.1016/j.immuni.2019.03.025 PMC6839410 30995506

[18] Rider CF Carlsten C. Air Pollution and DNA Methylation: Effects of Exposure in Humans. Clin Epigenet (2019) 11:131. doi: 10.1186/s13148-019-0713-2 PMC6724236 31481107

[19] Kohli A Garcia MA Miller RL Maher C Humblet O Hammond SK. Secondhand Smoke in Combination With Ambient Air Pollution Exposure is Associated With Increasedx CpG Methylation and Decreased Expression of IFN-γ in T Effector Cells and Foxp3 in T Regulatory Cells in Children. Clin Epigenet (2012) 4:17. doi: 10.1186/1868-7083-4-17 PMC3483214 23009259

[20] Tao R Cao W Li M Yang L Dai R Luo X. PM2.5 Compromises Antiviral Immunity in Influenza Infection by Inhibiting Activation of NLRP3 Inflammasome and Expression of Interferon-β. Mol Immunol (2020) 125:178–86. doi: 10.1016/j.molimm.2020.07.001 32717666

[21] Yamada T Horimoto H Kameyama T Hayakawa S Yamato H Dazai M. Constitutive Aryl Hydrocarbon Receptor Signaling Constrains Type I Interferon-Mediated Antiviral Innate Defense. Nat Immunol (2016) 17:687–94. doi: 10.1038/ni.3422 27089381

[22] Baraldo S Lokar Oliani K Turato G Zuin R Saetta M. The Role of Lymphocytes in the Pathogenesis of Asthma and COPD. Curr Med Chem (2007) 14:2250–6. doi: 10.2174/092986707781696573 17896974

[23] Thurston GD Balmes JR Garcia E Gilliland FD Rice MB Schikowski T. Outdoor Air Pollution and New-Onset Airway Disease. An Official American Thoracic Society Workshop Report. Ann Am Thorac Soc (2020) 17:387–98. doi: 10.1513/AnnalsATS.202001-046ST PMC7175976 32233861

[24] Bonato M Gallo E Bazzan E Marson G Zagolin L Cosio MG. Air Pollution Relates to Airway Pathology in Wheezing Children. Ann Am Thorac Soc (2021). doi: 10.1513/AnnalsATS.202010-1321OC PMC8641808 34004126

[25] Estrella B Naumova EN Cepeda M Voortman T Katsikis PD Drexhage HA. Effects of Air Pollution on Lung Innate Lymphoid Cells: Review of *In Vitro* and *In Vivo* Experimental Studies. Int J Environ Res Public Health (2019) 16:2347. doi: 10.3390/ijerph16132347 PMC6650824 31269777

[26] Alves MP Schögler A Ebener S Vielle NJ Casaulta C Jung A. Comparison of Innate Immune Responses Towards Rhinovirus Infection of Primary Nasal and Bronchial Epithelial Cells. Respirology (2016) 21:304–12. doi: 10.1111/resp.12692 26611536

